# SlimMe, a Chatbot With Artificial Empathy for Personal Weight Management: System Design and Finding

**DOI:** 10.3389/fnut.2022.870775

**Published:** 2022-06-23

**Authors:** Annisa Ristya Rahmanti, Hsuan-Chia Yang, Bagas Suryo Bintoro, Aldilas Achmad Nursetyo, Muhammad Solihuddin Muhtar, Shabbir Syed-Abdul, Yu-Chuan Jack Li

**Affiliations:** ^1^Graduate Institute of Biomedical Informatics, College of Medical Science and Technology, Taipei Medical University, Taipei, Taiwan; ^2^International Center for Health Information Technology, Taipei Medical University, Taipei, Taiwan; ^3^Department of Health Policy and Management, Faculty of Medicine, Public Health and Nursing, Universitas Gadjah Mada, Yogyakarta, Indonesia; ^4^Department of Health Behavior, Environment, and Social Medicine, Faculty of Medicine, Public Health and Nursing, Universitas Gadjah Mada, Yogyakarta, Indonesia; ^5^Center for Health Policy Management, Faculty of Medicine, Public Health and Nursing, Universitas Gadjah Mada, Yogyakarta, Indonesia; ^6^Taipei Medical University Research Center of Cancer Translational Medicine, Taipei, Taiwan; ^7^Department of Dermatology, Wan Fang Hospital, Taipei, Taiwan

**Keywords:** artificial intelligence, artificial empathy, chatbot, diet bot, weight loss management, virtual diet assistant

## Abstract

As the obesity rate continues to increase persistently, there is an urgent need to develop an effective weight loss management strategy. Nowadays, the development of artificial intelligence (AI) and cognitive technologies coupled with the rapid spread of messaging platforms and mobile technology with easier access to internet technology offers professional dietitians an opportunity to provide extensive monitoring support to their clients through a chatbot with artificial empathy. This study aimed to design a chatbot with artificial empathic motivational support for weight loss called “SlimMe” and investigate how people react to a diet bot. The SlimMe infrastructure was built using Dialogflow as the natural language processing (NLP) platform and LINE mobile messenger as the messaging platform. We proposed a text-based emotion analysis to simulate artificial empathy responses to recognize the user's emotion. A preliminary evaluation was performed to investigate the early-stage user experience after a 7-day simulation trial. The result revealed that having an artificially empathic diet bot for weight loss management is a fun and exciting experience. The use of emoticons, stickers, and GIF images makes the chatbot response more interactive. Moreover, the motivational support and persuasive messaging features enable the bot to express more empathic and engaging responses to the user. In total, there were 1,007 bot responses from 892 user input messages. Of these, 67.38% (601/1,007) of the chatbot-generated responses were accurate to a relevant user request, 21.19% (189/1,007) inaccurate responses to a relevant request, and 10.31% (92/1,007) accurate responses to an irrelevant request. Only 1.12% (10/1,007) of the chatbot does not answer. We present the design of an artificially empathic diet bot as a friendly assistant to help users estimate their calorie intake and calories burned in a more interactive and engaging way. To our knowledge, this is the first chatbot designed with artificial empathy features, and it looks very promising in promoting long-term weight management. More user interactions and further data training and validation enhancement will improve the bot's in-built knowledge base and emotional intelligence base.

## Introduction

Obesity is one of the leading causes of various chronic diseases, which may impair individual health, and leads to premature death (1). However, identifying effective strategies to significantly reduce body weight is still a significant challenge (2). The current behavioral weight loss programs that focus on self-monitoring of energy balance have proven to be the most effective way to lose weight (3). This tracking method relies on daily recording of individual calorie intake and calories burned from exercise. Despite its effectiveness, self-monitoring is known to be burdensome and has low adherence, which is thus associated with short-term weight loss (4).

Intensive behavioral treatments with prolonged interaction between patients with obesity and providers (*via* outpatient clinic or internet or telephone) combined with social support from the community are required to achieve a robust and effective weight loss intervention (5–7). Unfortunately, human dietitians cannot provide 24-h comprehensive monitoring and motivation to their clients (6). Given that humans have limitations, utilizing a virtual agent such as an AI chatbot can help overcome the lack of a human dietitian's 24-h monitoring or social support. Unlike traditional text-based messaging systems that still need a human operator to respond to a user message, chatbot technology enables users to have a real-time and engaging conversation with a computer as if they are talking to a human. This new communication platform has been shown to enrich the user experience and improve user engagement because it can quickly express empathy in response to user emotions, which is beneficial for successful long-term weight management (8, 9).

Emerging technology such as chatbot technology offers an excellent opportunity to assist humans with specific tasks in many fields (10). A chatbot is a computer agent that can interact with the user through a chat interface or messaging apps (such as LINE, Facebook messenger, and telegram). The first chatbot called ELIZA was developed in 1966 to impersonate human-to-human conversation by utilizing pattern matching based on a pre-determined conversational workflow known as rule-based chatbot (10, 11). Another notable chatbot Artificial Linguistic Internet Computer Entity (ALICE) adopted a pattern matching approach and was built using Artificial Intelligence Markup Language (AIML) technology (11). Although the ALICE architecture used a more extensive database than ELIZA, it still follows a rule-based pattern matching that lacks intelligent features and only generates a limited chatbot response (10, 11).

AI technologies, including machine learning, natural language processing, and intelligent analysis, have initiated big milestones in chatbot creation. This technology has enabled the chatbot to learn more conversational experiences and generate responses based on the previous history or chat interactions (11). While rule-based chatbots used pattern matching approaches and pre-defined conversational workflows to interact with the user, AI-based chatbots adopted a conversational AI to interact with the user through verbal (voice) and non-verbal commands (text, facial expression, gesture, and so on) (12). Apple Siri, IBM Watson, Google Assistant, Microsoft Cortana, and Amazon Alexa are among the most popular conversational AI that can serve as voice assistants (10, 11). Another conversational AI capable of engaging in conversation with humans using verbal and non-verbal communications in a robotic body or graphical front-end like avatars or cartoons is known as embodied conversational agents (12).

AI chatbots offer flexible conversation and persuasive technology to promote user engagement in chatbot activities (13). Flexible conversation means the chatbot can dynamically adapt to the user's emotional expression and engage in the chat interaction. Meanwhile, persuasive technology corresponds to how the machine can influence users' attitudes and behavior, enabling the bot to understand how humans naturally speak (14). Furthermore, recent advances in NLP using large pre-trained language models such as Bidirectional Encoder Representations from Transformers developed by Google (15), Generative Pretrained Transformer [GPT-2 (16) and GPT-3 (17)] by Open AI, and DialoGPT (18) and Turing Natural Language Generation (T-NLG) by Microsoft (19) offer a promising approach to create a human-like conversation that can understand specific user contexts and conditions. Moreover, some AI chatbots are also equipped with real-time sentiment analysis features to recognize user emotions. Once the chatbot analyzes the user sentiment, it will then direct cognitive responses such as empathic conversation and human–machine emotional bonding, potentially enhancing user experience and prolonging user engagement (9).

On the contrary, traditional rule-based chatbots communicate based on scripted conversation (11, 20). It follows a rigid conversational structure that is only capable of delivering correct responses when the user includes specific keywords programmed beforehand (20). This will limit the chatbot's approach to mimicking human conversation unless it is built with an extensive database, which is difficult to deal with. Although a rule-based chatbot can deliver emotional responses using keyword matching emotion lexicons and word embedding (21, 22), the responses generated seem unnatural and sound less human. It also has a slow response speed and cannot learn from past conversations, leading to a frustrating user experience.

With the development of AI-based chatbots and the rapid spread of the messaging platform and mobile technology with easier access to internet technology, chatbot innovation is widely adopted to support lifestyle behavioral changes during weight loss intervention. For example, Fadhil et al. (23) proposed an AI chatbot for nutrition education to promote healthy and sustainable eating to prevent weight gain in the adult population. A personalized virtual coach, “Denk je zèlf!” was also introduced by Dol et al. to support self-regulation of emotions in young obese emotional eaters (24, 25). Another example is Forksy, which monitors users' healthy eating behavior (13). A telegram chatbot Wakamola also serves as a valuable tool to assess individuals' obesity risk based on their sociodemographics, diet, physical activity, BMI, and social lifestyles (26). A preliminary review of AI chatbot-based physical activity and diet interventions revealed that more than half of the reviewed chatbots successfully deliver behavior change strategies through increased user motivation and engagement (27). Furthermore, users with high engagement levels tend to experience positive progress toward losing weight (27).

Although there are several AI chatbots developed to support weight loss management, a systematic review on AI chatbot interventions in changing physical activity, healthy eating, and weight management behaviors showed that, among nine studies included, more than half of the studies focused on promoting physical activity and only limited studies focused on a healthy diet and weight status (28). Moreover, other scoping reviews on AI chatbots for weight loss showed that the use of empathic chatbot functions in supporting a behavior change framework to guide AI chatbot development is still unclear due to a limited number of publications (9). As a result, less is known about incorporating chatbot ability to express empathy in developing an effective weight management program.

Given the rising trend of chatbot innovations in weight loss and physical activity promotion, it is critical to build a chatbot that can simultaneously understand the user's emotions and circumstances (27). Since losing weight can be emotionally and mentally demanding, receiving emotional support will benefit people who intend to lose weight (6, 29), especially when they feel discouraged. Although many apps serve as diet assistants (29), they mainly focus on dietary recommendations and calorie tracking with limited support for the user's emotional wellbeing (29, 30). Understanding individual emotions will positively impact the user's intention to follow a weight loss intervention (29).

This study aimed to propose an initial step to developing a diet bot with artificial empathy called “SlimMe.” Based on the proposed design, we conducted a preliminary evaluation to find bugs or errors and investigate how users react to SlimMe.

## Materials and Methods

This study proposed using an automated conversational agent *via* a social messaging platform that can supplement professional dietitians providing 24-h monitoring support to their clients (Supplementary File 1). Our chatbot is intended to support specific dietary weight loss approaches, focusing on self-monitoring energy balance, including self-weighing, dietary self-monitoring, and self-monitoring exercise.

### SlimMe Design Architecture

SlimMe is a conversational agent aimed to assist people who intend to lose or maintain their weight. It interacts with the users through text-to-text conversations *via* the messaging platform on their smartphone. The SlimMe chatbot architecture consists of dialogue management, messaging platform, and webhook services.

#### Messaging Platform

The messaging platform serves as the front end that provides the chat interface to interact with the bot. This study used LINE mobile API messenger platform as the chatbot interface. LINE is one of the fastest-growing messaging apps worldwide, with the number of daily new users reaching 1.7 million per day (31). In addition, the LINE platform supports integration with Dialogflow, allowing developers to develop LINE intelligence conversational bots with natural language understanding based on Dialogflow technology (7). Currently, SlimMe can be accessed by the LINE messenger with user ID @kze1036y or through a QR code image (Supplementary File 1).

#### Dialogue Management

Dialogue management is responsible for processing the user's message input and generating the appropriate response message (conversational agent). SlimMe uses Dialogflow as the conversational interface logic to manage the agent response. Dialogflow is a platform based on NLP provided by Google (32). This platform is free and supports multiple chatbot creations and language options.

SlimMe uses natural language understanding (NLU) to retrieve unstructured user input and generate an understanding context based on the user's intention. NLU supports intent classification and entity mapping (11). The intent is a concept that makes the chatbot understand and react to a specific action. An entity is a recognized concept (known as entities) within the custom intents (32). SlimMe intent classification and entity mapping model adopts a combination of pre-trained (manually annotated from specific text messages) and automated retrieval approach and generative response based on AI. The retrieval and generative-based approach uses artificial neural networks to compute all users' input and give probability scores for each intent using named-entity recognition (NER). We have created 103 intents with ~1,543 user expressions based on information knowledge from a professional dietitian and Nutritionix API database. In case there is no match response to the pre-defined set of rules, SlimMe will use the retrieval and generative-based approach to provide an appropriate response to the user's input in an open-domain conversation.

#### Nutrition Database and Webhook Services

Both Dialogflow and LINE platforms solely provide simple interaction with the user in the form of dialogue. We improved our bot knowledge with Nutritionix Food and Exercise Database API to add various nutrition consultation features. Thus, it could count users' daily calorie needs, estimate calorie intake, and burn calories after exercising. Nutritionix is the largest verified public food database with more than 800,000 unique food items and exercise data (33). In addition, Nutritionix API provides natural language for nutrient and exercise and is available on the Internet.

We utilized Heroku's webhook services based on Node.js for the backend response to set up a connection between SlimMe and Nutritionix. When the user adds our LINE bot as their friend or sends a message, it will post a notification to the webhook URL and call a pre-defined function. Once the action is completed, the appropriate responses will be selected and sent to the user *via* the LINE messenger.

The chatbot knowledge models were built by adopting the self-monitoring behavior that records user self-weigh-in, activity, and persistent food logging. Self-monitoring behaviors were known to be significant predictors of weight loss (34). As a virtual diet assistant, SlimMe enables the user to experience simulation on self-monitoring behavior features such as nutrition assessment, food intake, and exercise history. Nutrition assessment features collected basic user profiles that can calculate the user's daily calorie needs. Food intake features can estimate the user's daily calorie intake. Meanwhile, the exercise feature collected information on the user's exercise activity to estimate calories burned after exercise.

**Figure 1:**
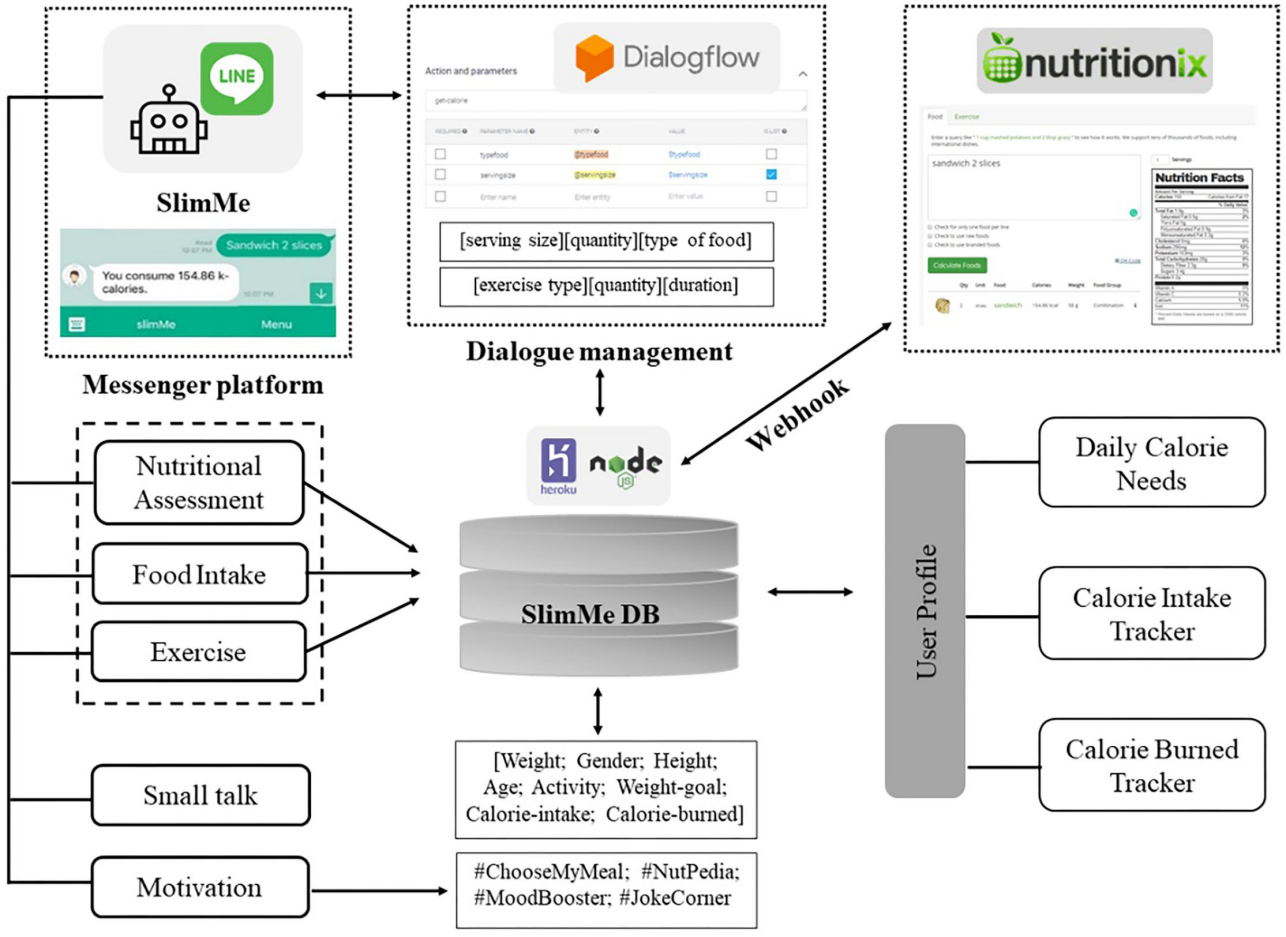
SlimMe architecture and knowledge-based model.

Figure 1 illustrates how we built SlimMe chatbot information knowledge based on user expression responses. First, the inputted messages related to nutrition features, food intake, and exercise features will be extracted and stored in the SlimMe database. Next, user information such as weight, height, age, gender, physical activity level (PAL), and weight goal will be extracted to estimate the user's daily calorie needs, known as total energy expenditure (TEE). Finally, TEE calculates the individual basal metabolic rate (BMR) based on the Harris–Benedict Equation (35). A detailed explanation of the TEE and BMR calculations can be seen in Supplementary File 2.

In addition to self-monitoring behavior, personalization through individualized feedback provided by coaches offers an opportunity to increase participant engagement and be a significant predictor of greater weight loss. Therefore, we offer empathic motivational support, which consists of a self-report reminder of the user's food intake and exercises (#ChooseMyMeal), nutritional knowledge (#NutPedia), inspiration quotes (#MoodBooster), and even jokes (#JokeCorner). We also provide small talk features to respond to any messages that are not related to nutritional content. This small talk knowledge base was built using a hybrid of pre-defined conversations on weight loss and pre-built agents for real-world conversation and the automated retrieval and generative-based AI model on the user input history in Dialogflow.

We adopted a text-based emotion analysis to recognize the user's emotion. We provided the bot with a dictionary of positive and negative emotions from a series of conversational flows from a pre-built agent in Dialogflow combined with machine learning training based on the user interaction history. Examples of positive emotions are “happy,” “good,” etc. and negative emotions are “sad,” “angry,” “bored,” etc. These dictionaries enabled SlimMe to detect positive and negative emotions and then provide appropriate responses regarding such emotions. SlimMe generated a persuasive messaging response using the right choice of words to express the feeling of understanding. We optimized the bot's empathic expression by adding social dialogue (small talk features), knowledge (motivational features), humor, and media (e.g., messaging stickers, emoticons, images, and video) that can emulate human-like conversations.

**Figure 2:**
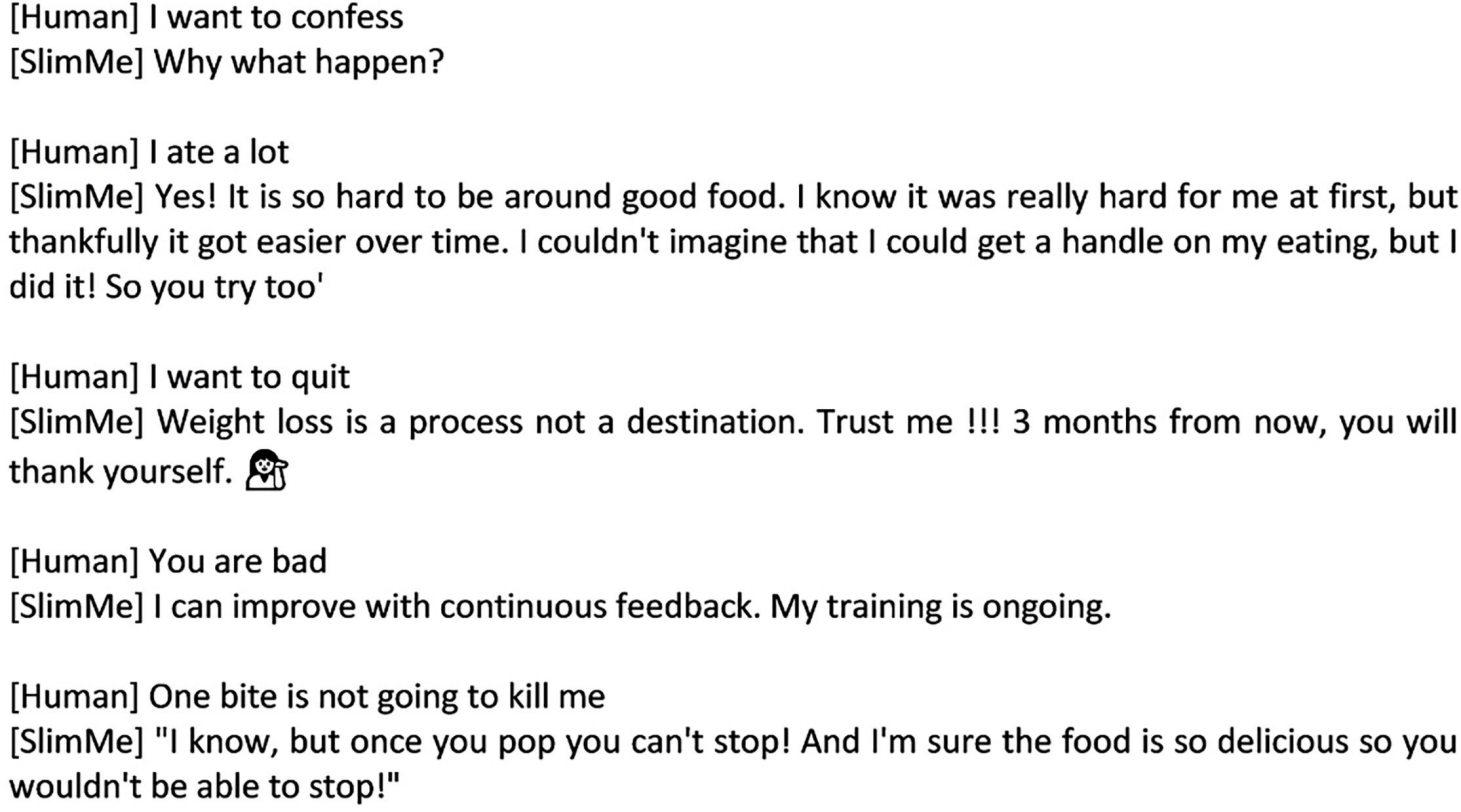
Sequence conversation between SlimMe and users.

The sample conversation sessions between a user and SlimMe can be seen in Figure 2. When users encounter an emotional situation, SlimMe treats the user as a friend and gives them a chance to express their current emotional status. If users express their feelings, SlimMe responds positively to understand their situation before providing motivational support.

### Participant and Procedure

We recruited 10 international students at Taipei Medical University for our simulation trial through convenience sampling. We selected early-stage users as representative samples who were not programmers and not aware of or involved in the development of SlimMe. They were also new to the concept of diet bot. They should have prior experience using mobile nutrition diet apps, so that they are familiar with the self-nutrition reporting process. In addition, we only included female participants because women are more likely to be engaged in the health promotion program and more conscious about body weight. Women were more likely than men to report using an organized weight loss program during their weight loss (36), which is really important to improve our chatbot daily calorie and exercise trackers. We asked all participants to interact with SlimMe every day for 7 consecutive days. SlimMe also sent a reminder two times a day to remind the participants to report their dietary intake and physical activities. We provided a shopping coupon as a small reward to the participants at the end of the trial.

We asked the participants to fill out a short questionnaire about demographic characteristics, smartphone usage, and familiarity with chatbot programs before the 7-day simulation trial. The participants were then asked to add SlimMe as their LINE friends. We then informed the users on how to interact with the bot and provided them with a simulation scenario to train the chatbot function. The simulation scenario was divided into three different tasks (Table 1), based on the complexity of the NLP and nutrition task: (1) simple task is contributed to any daily conversation related to weight loss; (2) complex task is defined as a specific nutritional assessment conducted using a rule-based approach that will follow a sequence of conversations in which the chatbot will record the information and then calculate the recommended dietary calorie restriction based on the user self-report assessment; and (3) very complex task is involving providing self-report of any dietary intake and exercise, in which the chatbot will record the information provided and search in its nutritional database and then calculate the calorie based on the user's self-report.

**Table 1:** SlimMe chatbot simulation scenario.

**Task**	**Item**	**Explanation**
Simple	Greetings	Try to greet the bot
	Small talk	Inform the bot that you want to lose weight, struggle with your diet, food pusher comment, say thank you, say the bad appraisal
	Asking bot identity	Try to ask [what is chatbot] and [what it is capable of], who develops the bot, etc.
Complex	Diet goal	Select the diet plan offered by the bot
	Nutritional assessment	Inform your weight, gender, height, activity level, and weight goal
	Daily calorie needs	Ask your daily calorie need
VERY Complex	Calorie intake tracker	Inform the bot what you eat and portion and ask the bot the calculate the calorie
	Calorie burned estimation	If you manage to do some exercise, ask the bot to estimate how many calories does it burn by providing the type of exercise and the duration
	Calorie log	Track your calorie need, intake, and burned from exercise

### User Experience Evaluation Measurement

After the simulation session ended, we asked the participants to answer a questionnaire to investigate their experience. We used a five-point Likert scale questionnaire (ranging from 1 for “strongly disagree” to 5 for “strongly agree”), adopted from the chatbot evaluation questionnaire introduced by Quarteroni and Manandhar (37) and chatbot user experience evaluation by Duijst et al. (38). We reversed the score for each negative statement. We instructed the participants to indicate their agreement level related to SlimMe performance (four items), usability (four items), usefulness (six items), and satisfaction (four items) as described in Supplementary File 3. We asked the participant to provide their comments and feedback about their experience and interaction with the SlimMe chatbot using four questions: “What did you think of this experience? Why (not)?,” “Do you think you would use this chatbot in real life? Why (not)?,” “Do you think this chatbot is useful? Why (not)?,” and “Did you think functionalities were missing in the chatbot? Which one?”. We also encouraged the participants to capture any problems they encountered during their interaction with SlimMe. Feedback was then analyzed to identify further needs to revise the chatbot prototype.

In addition to evaluating the user experience, we also described the chatbot evaluation metrics to obtain an objective result related to the user interaction frequency with SlimMe during the 7-day simulation period. First, we recorded all conversation history using Dialogflow and counted the total number of bot responses and the total user input messages recorded. Dialogflow can flag out chatbot irrelevant responses by enabling the machine learning process. We then manually reviewed all responses and classified them into coherent and incoherent responses. The coherent response indicates an accurate bot response to a relevant user request. Meanwhile, the incoherent response indicates an inaccurate bot response to a relevant user request. We defined relevant user requests as any user input that related to the weight loss domain as instructed in the simulation scenario.

### Statistical Analysis

Reliability testing was conducted to measure the internal validity and consistency of items used for each construct variable, such as performance, usability, usefulness, and satisfaction. In addition, Cronbach's α was calculated to measure the internal consistency of the questionnaire. All statistical analyses were performed using R software (version 4.0.5). A visualization of Cronbach's α is generated using the function alpha() from R package psych (39).

## Results

### SlimMe User Interface

**Figure 3:**
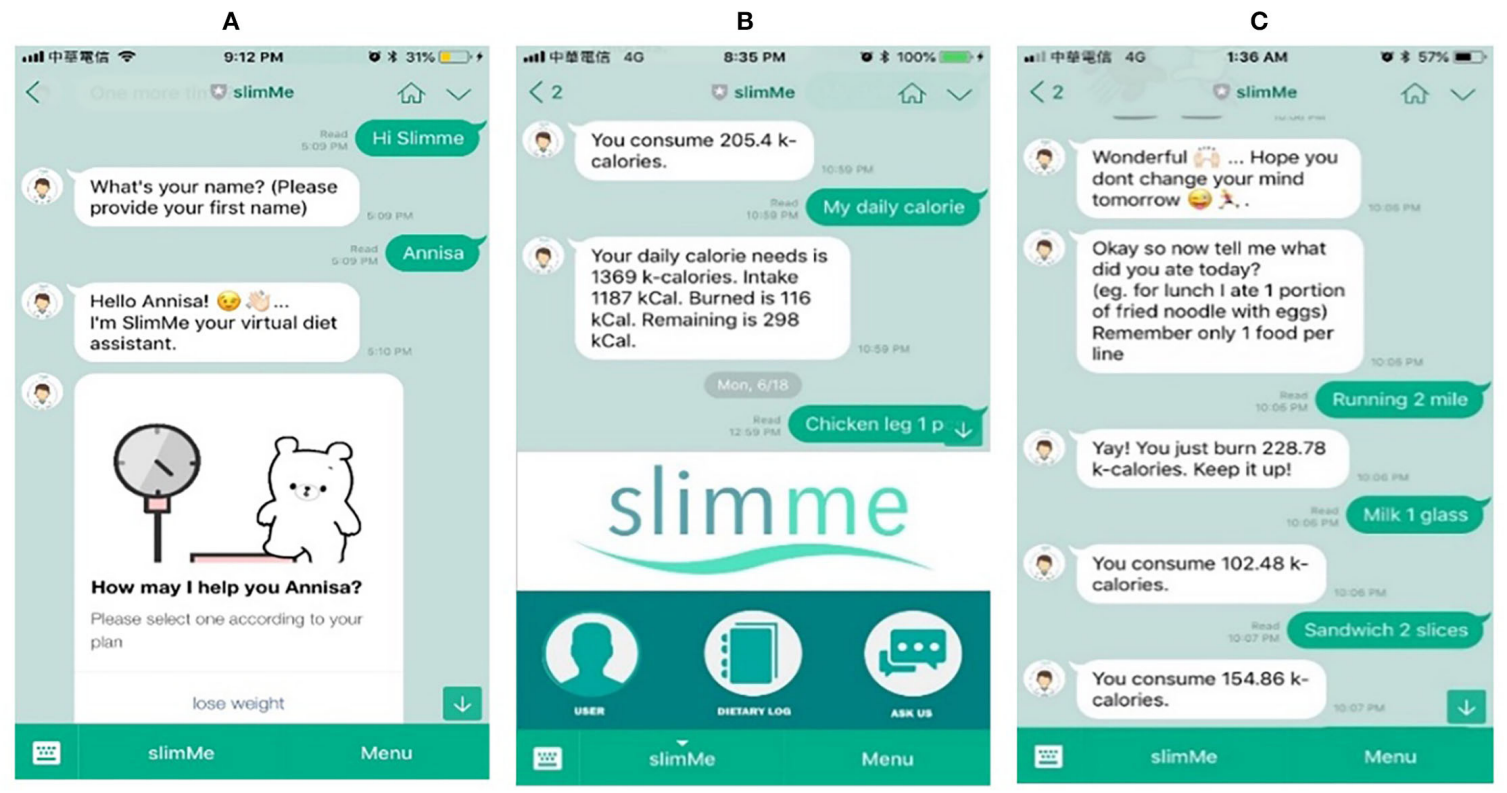
SlimMe design interface using LINE messenger platform. **(A)** Greeting to start the nutrition assessment. **(B)** Daily calorie record. **(C)** Calorie intake and exercise tracker.

Figure 3 describes the nutrition assessment simulation features. For example, if a user informs the bot that the user wants to lose weight (Figure 3A) and agrees to join the dietary planning, the SlimMe bot will ask about the user's age, gender, weight, height, and physical activity. Then, the bot will estimate the user's daily calorie need based on the user information profile and the user's desired weight goal (Figure 3B). The bot will also estimate the user's daily calorie intake based on the self-reported input (Figure 3C). To estimate the user's calorie intake, the user can type the food name and the portion size, and SlimMe will present the calorie and record it in the dietary log. The user can also inform SlimMe the type of exercise they did, the duration, and the number of calories they burned (Figure 3C).

SlimMe chats interactively with the users by creating a responsive and interactive response through motivational features (Supplementary File 1). It reminds the users to inform their calorie intake and exercise activities and motivate and provide nutritional knowledge, inspirational quotes, and even jokes. Furthermore, SlimMe expresses its empathy through various emoticons and GIF images, emulating a human-like chatting.

### SlimMe Chatbot Persuasive Technique and Behavior Change Model

SlimMe adopted a persuasive technique to deliver empathic and engaging conversation flows to the user. This persuasive mechanism targeted two significant activities, namely, general conversation to maintain the conversational flow and affective conversational capacity with empathic and persuasive messages to change user behavior (27). To achieve a successful weight loss intervention program, we implemented a comprehensive behavior change model focusing on energy intake reduction, physical activity improvement, and behavioral skill training (40). We adopted the behavioral change wheel (BCW) (41) associated with the AI chatbot behavioral change model (27) as the framework for the chatbot development. The BCW model includes three sources of behavior, namely, capability (a lack of knowledge about the benefits of AI chatbot services to promote healthy diet and lifestyle modification), opportunity (the possibility of an AI chatbot to provide artificial empathy based on persuasive technology to change the user's behavior), and motivation (encourage positive feelings, affective feedbacks, and achievement rewards). We linked the BCW model with the AI chatbot behavioral change model divided into four major components: (1) defining chatbot characteristics (e.g., SlimMe visual illustration, identity, and features) and user backgrounds (sociodemographic characteristics, nutrition assessment, and self-reported dietary intake and physical activities), (2) building relational capacity (small talk features and learning the user's chat interactions), (3) building persuasive conversational capacity (nutrition and exercise knowledge database, persuasive strategies using pre-built emotional appeals and affective messages), and (4) evaluating mechanisms and outcomes (early-stage user experience and usage patterns).

### Result From the Simulation Trials

All of the simulation trial respondents were women, with age ranging from 24 to 34 years, and half of them have nutrition and dietetics as their educational background. The mean BMI was 22.24 kg/m^2^ (normal weight). Among the participants, five (50%) were overweight, four (40%) were normal weight, and one (10%) was underweight. Of these, 70% were Android smartphone users, and half of them had previous experience with chatbots other than SlimMe chatbot before. Apart from participants' characteristics of using a smartphone regularly, the messaging platform ranked fifth in favorite smartphone apps.

### Chatbot User Experience Evaluation

**Figure 4:**
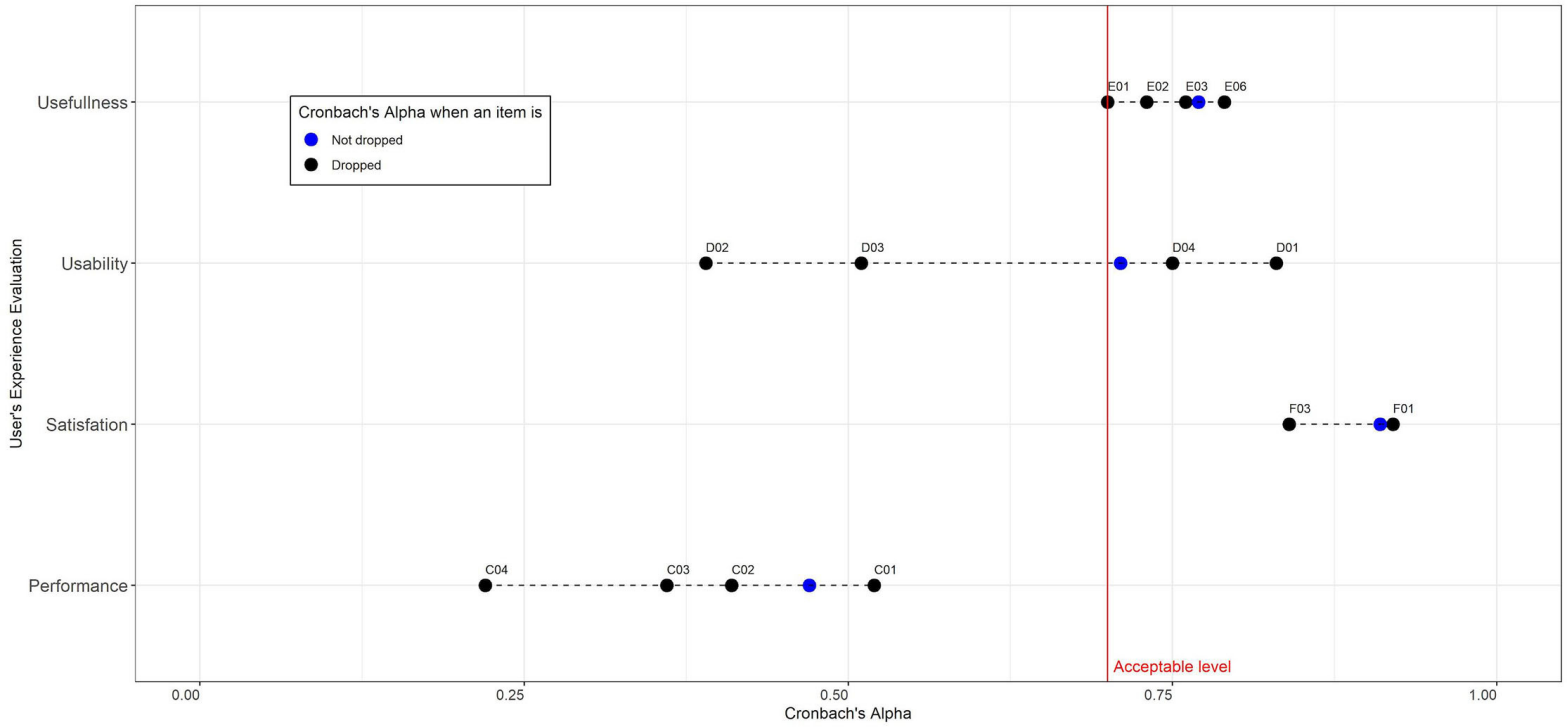
Distribution of Cronbach's α within the four constructs of user experience evaluation.

Figure 4 illustrates a visualization of Cronbach's α distribution. The Y-axis shows the four constructs of user experience evaluation; meanwhile, the X-axis illustrates Cronbach's α value (range from 0 to 1) for each item within the four constructs on the Y-axis. Every item in each construct is represented as a horizontal line with multiple dots. The points in blue color indicate Cronbach's α value for the overall items in each construct, and the points in black color indicate Cronbach's α value when one of the construct items is dropped.

Based on Cronbach's α analysis, the four construct user experience variables were varied for their reliability, the construct usability (*n* = 4 items, α = 0.71), the construct usefulness (*n* = 6 items, α = 0.77), and the construct satisfaction (*n* = 4 items, α = 0.91). All three constructs had a value greater than the minimum acceptable level of 0.7 (red vertical line), indicating that the constructed variable was reliable and well-constructed. Meanwhile, the construct performance's internal consistency is below the acceptance level (α = 0.47, *n* = 4 items). We have considered dropping one item (C01) that contributed to such low reliability for the performance construct and achieved acceptance level (α = 0.54, *n* = 3 items). For all four constructs, the α value indicated high reliability of statements (α = 0.89). The detailed description of the user agreement for the four construct variables can be seen in Supplementary File 4.

The result from the chatbot evaluation metrics showed there were 1,007 bot responses from 892 user input messages. In addition, there were 601 (67.38%) coherent response messages and 189 (21.19%) incoherent response messages. Moreover, we noticed that SlimMe provided an accurate response message 92 times (10.31%) to an irrelevant user request, and only 10 times (1.12%) did the bot fail to respond to user messages.

The user feedback evaluation results about user experience with SlimMe showed three key themes: positive chatbot user experiences, negative chatbot user experiences, and future use.

#### Positive Chatbot User Experiences

The most frequent positive chatbot user experiences involved ease of use, usefulness, and fun attributes. The user indicated that the SlimMe chatbot is very useful because it is easier to track their calorie intake and calories burned without having new apps installed on their smartphone. Having a chatbot talk to them about diet plans and estimate their daily calorie needs is a fun and exciting experience. The users felt positive about the use of emoticons, stickers, and GIF images. They noticed that this function makes the conversation more interactive.

“*It's so fun to use this chatbot since it helps me to count my input and output calories.”* [Participant 3]

“*Great! It looks like I have a personal assistant.”* [Participant 10]

Having the chatbot remind them two times a day to report the food they ate and the exercise they did is acceptable for them. Most users noticed that some nutrition knowledge, motivation, and even some jokes are fresh and fun and make the chatbot more empathic.

“*I like it when it reminds me about what I've eaten the whole day from its question. When I report whatever food/beverages I take, I always have a guilty feeling than suddenly think, ‘I will take less later*.” [Participant 9]

#### Negative Chatbot User Experiences

We highlighted negative chatbot user experiences contributed by the calorie tracker feature and time limit setting. First, users indicated that they want the chatbot to remind them at least three times a day during meal times to be more accurate to them as if they are having their friends care for them.

“*Yes, but sometimes chatbot doesn't remind me at every mealtime, so sometimes I forget.”* [Participant 7]

Second, dietary calorie needs can only provide information on the daily calorie summary. The user expected the bot to inform the calorie and macronutrient information, such as carbohydrate, fat, and protein, weekly or monthly.

“*I think maybe providing a note/summary of what keywords are recognized by the system can improve the function a lot. Also, maybe at the end of the day, we can get a notification of the summary, how many calories we eat.”* [Participant 5]

“*Yes. I think the developer can increase other nutrition components such as vitamins, protein, and fat. In addition, the chatbot can give the consumer a daily report for the intake and burned calories at the end of the day for each day.”* [Participant 8]

Setting up a time limit during sequence conversation will become an obstacle to the users during the simulation. We are aware that sometimes the users cannot reply to the bot directly, and when the users have time to reply, the bot can no longer recognize the users' expression due to the time limit response.

“*The time is a bit annoying. When I was too late to reply, then the chatbot couldn't recognize what I typed. The other thing is the measurement scale.”* [Participant 9]

#### Future Use

When asked whether they wanted to use the SlimMe chatbot in real life, all the participants responded positively.

“*I will recommend this chatbot to my patients & fellow dietitian.”* [Participant 1]

“*Yes, because it helps me track my calorie needs/burn in a simple way.”*[Participant 4]

Based on the chat interaction history between the chatbot and the user, we found that the user tends to forget to inform the bot about the portion size or input a wrong information order, so SlimMe cannot provide an appropriate response. For example, the user only typed “1 (banana)” without providing the serving size, or instead of writing “1 (glass) of (milk),” they type “milk one glass.” The lack of an entity dictionary caused this issue. Initially, the bot can only estimate the user's calorie intake by recognizing the sequence order of (1) portion size and then (2) the food type [e.g., (1 cup) of (rice)]. Therefore, we added a fallback intent with illustrative chat examples on the calorie tracker functions and updated the entity dictionary with unrecognized serving sizes to resolve this issue.

In addition to the calorie intake tracker issue, the bot also failed to estimate the calorie burned from exercise because the user did not provide the correct exercise type. For example, users type unrelated activities such as reading a book or studying as their exercise type, instead of typing sitting and asking the bot to estimate the calorie burned. However, because we only trained the chatbot with a certain unit of time (e.g., second, minute, and hour) and distance (e.g., mile, km, and m), the chatbot failed to estimate the calorie burned if the user used a new entity other than the one input in the dictionary. For example, SlimMe was unable to recognize an inputted variable “steps” as their length duration. Therefore, we tried to improve the bot intelligence by adding unrecognized exercise type information such as “steps” in the calorie burned tracker dictionary. A conversational assistant's performance depends on the dictionary model, especially when the bot provides an appropriate response to the user's various inputs. Therefore, we need to thoroughly validate every possible scenario provided by the user (21). We also tried to prevent errors by sending an example pattern to show how the user should inform the bot about their dietary intake and exercise activities. Dialogflow also provides a training module where the developer can look upon the mismatched conversation between user requests and chatbot responses.

We found some bugs during the simulation trials, especially when the user tried the dietary log function. Instead of generating the dietary log directly, the bot will send the latest calorie intake estimation and then provide the requested dietary log after the user re-clicked the menu function. We fixed the issue by disabling the default response in Dialogflow and enabling the webhook function's fulfillment response. Furthermore, our simulation trial respondents had a different ability to accurately estimate their intake portion size. They seem to have difficulty converting the portion size to gram or kilogram. Therefore, we would like to provide the portion size estimation cheat sheet as SlimMe's additional feature in the future. These findings probably affected the user's reaction to the performance of the chatbot.

## Discussion

### Principal Findings

This study was conducted to design a chatbot with artificial empathy to provide motivational support for a weight loss program and investigate early-stage user reactions to a diet bot. Our proposed conversation model that combines a pre-defined dialogue system and automated retrieval approach and generative response based on the user interaction history improved either bot conversation knowledge or nutrition and exercise knowledge. Improving the bot conversation capabilities based on the user interaction history enabled SlimMe to provide flexible conversation that adapts dynamically according to the user's emotional expression (12). Furthermore, affective AI conversational abilities supported with empathic and persuasive messages can build trust and correspond to the change in users' attitudes and behavior during weight loss intervention (27). A study reported that patients with high engagement levels with a behavior change chatbot have a relevant weight loss and improved diet (27).

Several studies have demonstrated a consistent correlation between successful long-term weight loss interventions and a self-monitoring diet in adjunct with motivational support (3, 4, 42). A randomized clinical trial of behavioral treatment for weight loss study reported that participants having a personal digital assistant with a daily feedback message group experienced a greater self-monitoring adherence with the greatest weight change (43). Therefore, an intervention that provides real-time and personalized emotional support, for instance, AI chatbots, yields a promising approach to prolonging the efficacy of weight loss intervention. However, this requires the chatbot to enhance its ability to have a more accurate human-like conversation, especially when users experience negative emotional feelings during a vulnerable situation in their weight loss efforts (9). A previous systematic review of chatbot intervention for weight management showed no valid evidence of the efficacy of chatbot intervention for weight loss (28). Yet, we believe that the application of AI chatbots for sustained weight loss will exhibit strong growth. Therefore, further study is required to improve user experience and evaluate the usability and effectiveness of SlimMe in supporting long-term success in weight loss management.

The result from the 7-day simulation trial indicated that, among the construct variables of usability, usefulness, and satisfaction, the usability construct statement items showed the least consistency with each other. It is probably related to the calorie intake and calorie burned tracker feature. Chatbot time response, which indicates the time interval between user input and the bot response, is also a key factor that affects user perception of the chatbot performances (44). SlimMe will respond to users in ~ <1 s. However, the descriptive summary results indicated that half of the participants experienced slow response time when interacting with SlimMe. After observing the interaction history, we noticed a slow response when the chatbot displays GIF images and estimates the calorie intake and calories burned from exercise. Several aspects may affect chatbot response time, such as network connection, chatbot server capabilities, load response, and smartphone processing response (44).

Another key metric that should be measured to evaluate the bot NLP model is the interaction frequency between the bot and the user at a certain time (38). The number of coherent and incoherent bot responses for each user input sentence can also reflect how relevant is the bot response for each question and how it matches the expected answer in the knowledge dictionary (37). The results from the chatbot interaction metric showed that we need to improve the accuracy of the SlimMe chatbot's response to the relevant user input request. We can enhance the chatbot's ability to predict the possible responses by validating the intent classification and entity recognition based on the past inputs of training data. Given the complexity of a real-world conversation, continuous training of our SlimMe chatbot will be needed. Although we had to focus the chatbot conversation on diet and exercise, the users were still demanding other conversational intents beyond the chatbot functions, such as small talk and advice. Therefore, we provided structured conversations with predictable input options to avoid infinite possibilities of user inputs. Meanwhile, the number of SlimMe chatbot inaccuracies to respond to relevant user input requests increased along with the increasing calorie intake and calorie burned tracker response. Based on the user interaction history, we found some repetitive tasks due to the bot's limitation of knowledge and users' lack of understanding to inform the bot of their intake portion size that we can address in future maintenance and development.

The simulation trials' results indicated that having a virtual agent that becomes a friendly assistant in the weight loss program is a fun and exciting experience for participants in terms of chatbot empathic features. A future extension to the proposed architecture may be needed for a better user–bot interaction, such as improving the SlimMe knowledge-based model (motivational features) and persuasive and empathic features. SlimMe knowledge of persuasive messages can be improved through speech recognition functionalities. Meanwhile, empathic features can be improved by enabling food image recognition. The user can upload the food image they consumed, and SlimMe will estimate their calorie intake. Due to users' confusion related to portion estimation in the simulation reports, it is also important to provide portion size and exercise duration information intent, which will help the users estimate the portion size and duration time. Automatic mass unit conversion should also be considered to estimate a more valid calorie intake based on household portion size. Through this approach, we believe it will minimize the users' difficulty to estimate the portion size.

In addition, the chatbot's attempts to express empathic functionalities might be slightly different from humans in conversations and probably appear inauthentic to the user. However, text-based emotion analysis offers a promising ability to detect users' emotions and respond appropriately based on the user statements. For example, in case the user indicates a negative feeling or emotion in their messages (such as “sad,” “angry,” and “tired”), the bot will react appropriately by using the right choice of words to express the feeling of understanding and help the user feel better (such as “I know,” “I understand,” telling jokes, and changing dialogue). We also optimized the bot's empathic expression by adding media (e.g., messaging stickers, emoticons, images, and video) to be friendlier and more emotionally intelligent. A large-scale language model on human-to-human conversations on weight loss and emotion should be incorporated into the training data sets to improve the chatbot's empathic ability. Training with large data sets, SlimMe can automatically generate a natural response that offers personalized support, exceeding its pre-defined functionalities. Meanwhile, to reduce the harmful behavior due to the chatbot malfunctions, developers need to provide shortcoming feedback and provide options to ensure an appropriate response to the user request. Continuous training is also needed to improve the chatbot's conversational capabilities in delivering empathy and mimicking human conversations.

### Limitations

This study has some limitations. First, the nutrition assessment methods such as the anthropometric data, dietary intake, and exercise were based on the user's self-reports, which may not be reliable and biased. Compared to the direct measurement, self-reported anthropometric measurements were less accurate (45) because people tend to under-report weight and over-report height (46). However, evidence revealed that self-reported assessment is satisfactorily valid for dietary and nutritional status measurements (47, 48). Second, our chatbot was currently only supporting English language conversation. However, our NLP logic platform can support multi-language conversation for ~16 languages (32). Thus, after some improvement in the nutrition and physical exercise database translation, it is possible to build a multi-language bot. Third, this study focuses on developing and deploying a diet bot, whereas the preliminary evaluation only involves a small number of female users. We intended to recruit specific users with domain knowledge of medical and nutrition before scaling up to a broad range of users as they are more prominent and relatively aware of the pros and pitfalls of diets. After some improvements, we aim to conduct a future study on comprehensive chatbot user experience, targeting individuals who are overweight and obese to compare the effectiveness of traditional weight loss and chatbot interventions. Furthermore, our chatbot is still using a text-based emotion recognition system, so there is room for improvement in the chatbot's performance and capabilities using image-to-text- and speech-to-text-based recognition systems.

## Summary

This study proposed the new approach of combining individual dietary intake and physical activity self-reports with emotional support using an artificially empathic chatbot that can deliver a fun, personalized, and effective weight loss management. We identified an architectural design of a diet bot composed of the dialogue management (the bot logic), messaging platform, and webhook services platform. The webhook services can improve SlimMe intelligence in responding to the user's calorie intake and calorie burnt that dialogue management cannot support. Moreover, the chatbot may express engaging responses by enabling a custom payload based on text-based emotion analysis in our NLP platform and learning the user communication history. Later, this framework can be adopted to build another virtual agent which supports other specific diet therapy. The chatbot can provide a real-time diet recommendation based on the user' dietary preferences. It can also synthesize a personalized diet based on user diet therapy target and their current achievement and emotional states.

## Data Availability Statement

Publicly available datasets were analyzed in this study. This data can be found here: https://github.com/annisaristya/slimme.

## Ethics Statement

This study has been approved by TMU Joint Institutional Review Board. Participant indicates their consent by accepting the simulation trial link invitation via LINE messenger.

## Author Contributions

AR was the primary investigator of this study, had the full access to all data, system in the study, and took responsibility for the integrity of the data and system. AR collaborated with MM to design the chatbot platform. BB and SS-A contributed to the chatbot UI/UX design. AR and AN were responsible for data collection, analysis, and visualization. H-CY and Y-CL contributed to study design, supervised the study, and obtained the funding. All authors read and approved the final manuscript.

## Funding

This study was financially supported by Beasiswa Unggulan Scholarship funded by the Ministry of Education and Culture, Republic of Indonesia, Ministry of Education (MOE), ROC Taiwan (grant number DP2-111-21121-01-A-02), and the Ministry of Science and Technology, ROC Taiwan (grant numbers: MOST 110-2320-B- 1222 038 -029 -MY3, 110-2221-E-038 -002 -MY2, and 110-2622-E-038 1223 -003 -CC1).

## Conflict of Interest

The authors declare that the research was conducted in the absence of any commercial or financial relationships that could be construed as a potential conflict of interest.

## Publisher's Note

All claims expressed in this article are solely those of the authors and do not necessarily represent those of their affiliated organizations, or those of the publisher, the editors and the reviewers. Any product that may be evaluated in this article, or claim that may be made by its manufacturer, is not guaranteed or endorsed by the publisher.

## Abbreviations

AI: artificial intelligence
BCW: behavioral change wheel
NER: named-entity recognition
NLP: natural language processing.
