# Supplementary material for: SlimMe, a Chatbot With Artificial Empathy for Personal Weight Management: System Design and Finding
Source: Front Nutr. 2022 Jun 23;9:870775. doi: 10.3389/fnut.2022.870775 (PMC9260382; doi:10.3389/fnut.2022.870775)
Supplement: Supplementary file 1 [file Data_Sheet_1.pdf]

Supplementary Material

Supplementary File 1. SlimMe User Interface

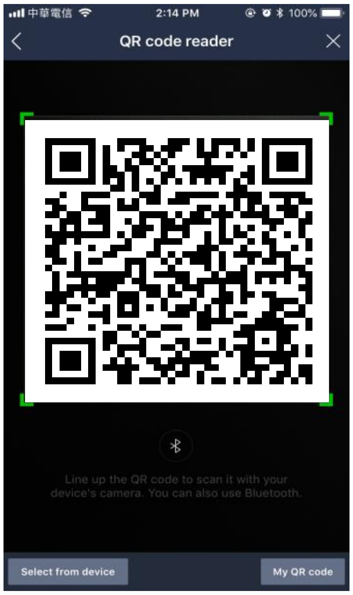

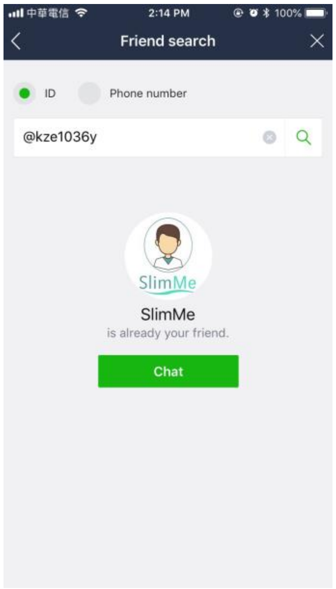

Social dialogue

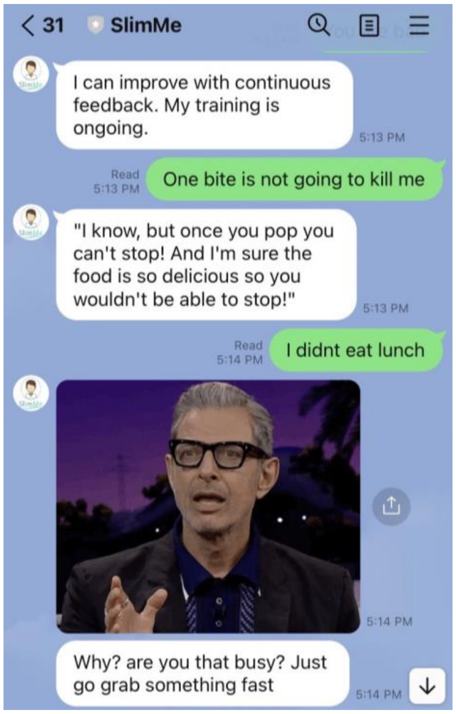

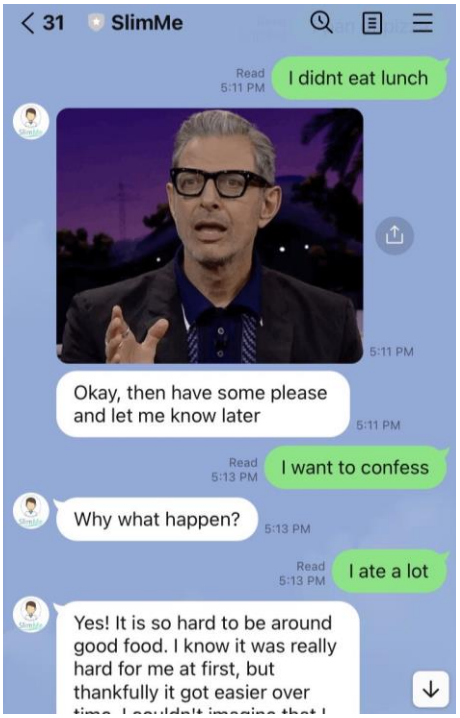

Motivational Features

Self-report reminder of the user's food intake and exercises (#ChooseMyMeal)

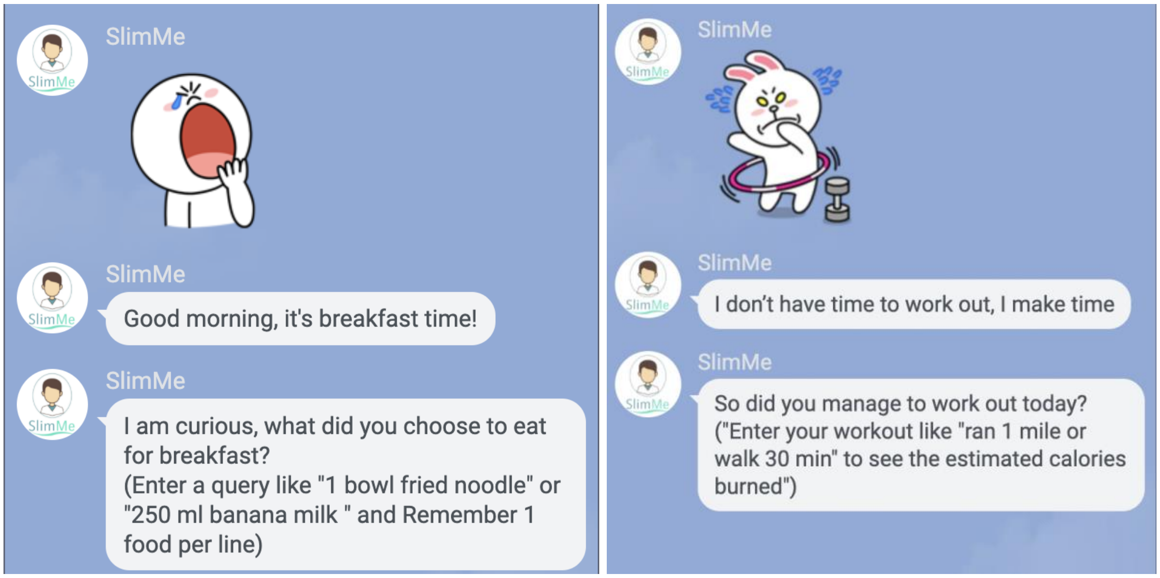

Nutritional knowledge (#NutPedia)

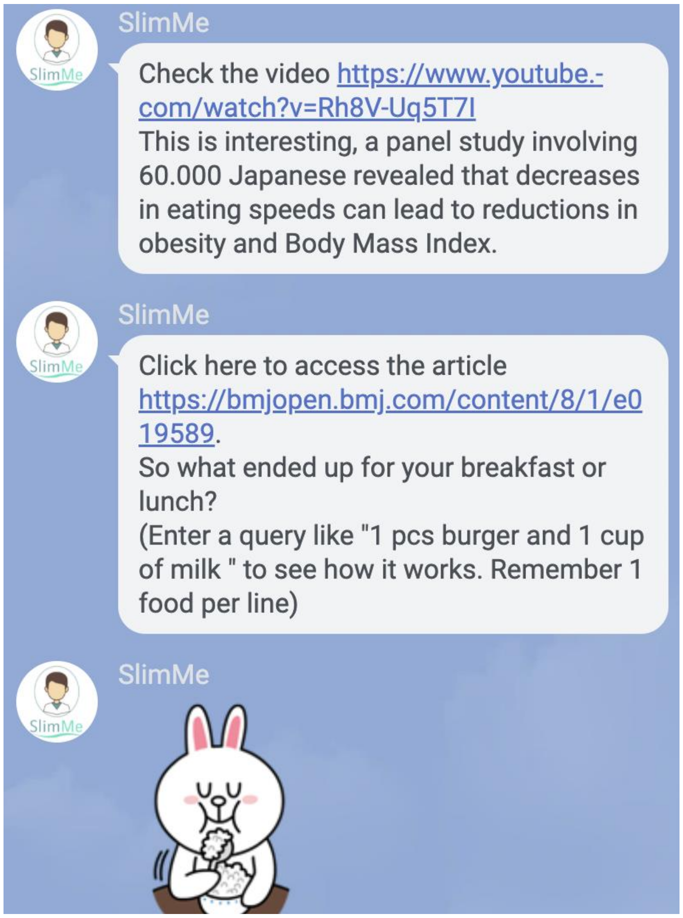

Joke (#JokeCorner)

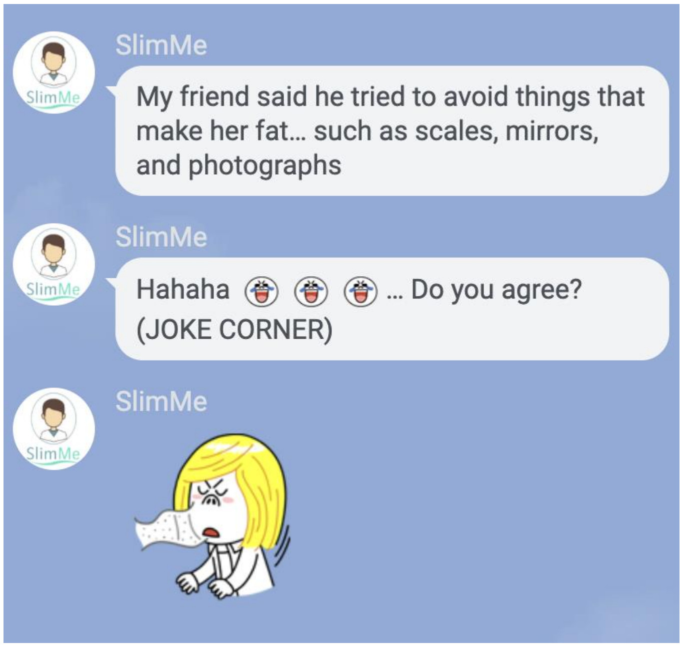

Inspiration quote (#MoodBooster)

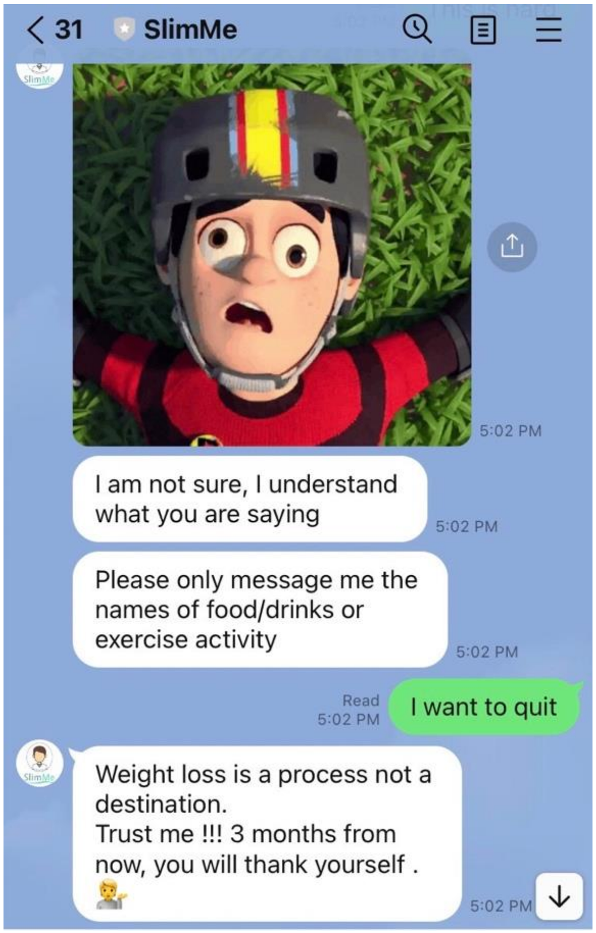

Diet Plan

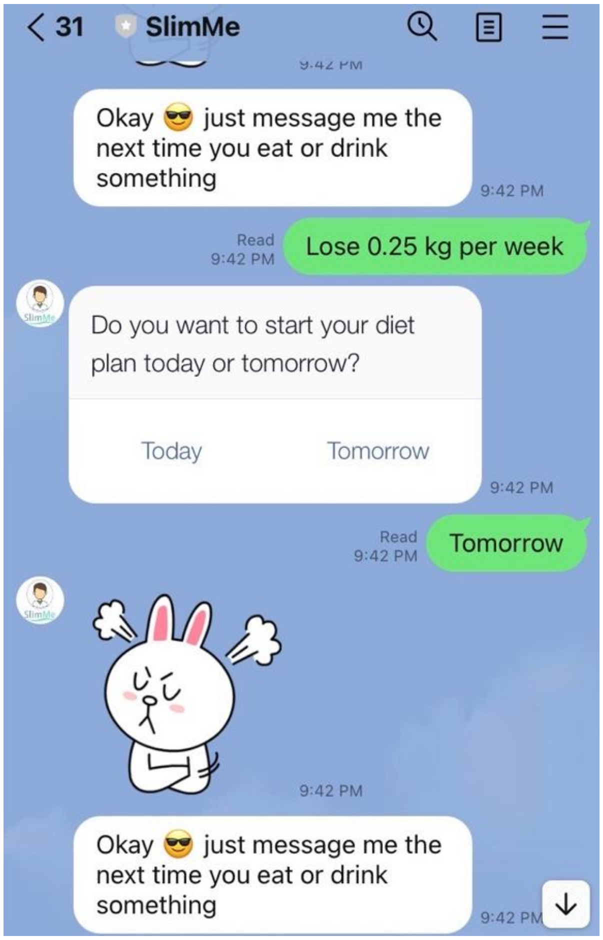
31
SlimMe
9:42 PM
Okay 😎 just message me the next time you eat or drink something
9:42 PM
Read
9:42 PM
Lose 0.25 kg per week
Do you want to start your diet plan today or tomorrow?
Today
Tomorrow
9:42 PM
Read
9:42 PM
Tomorrow
9:42 PM
Okay 😎 just message me the next time you eat or drink something
9:42 PM
