# Supplementary material for: SlimMe, a Chatbot With Artificial Empathy for Personal Weight Management: System Design and Finding
Source: Front Nutr. 2022 Jun 23;9:870775. doi: 10.3389/fnut.2022.870775 (PMC9260382; doi:10.3389/fnut.2022.870775)
Supplement: Supplementary file 2 [file Data_Sheet_2.pdf]

Supplementary Material

Supplementary File 2. Total Energy Expenditure (TEE) and Basal Metabolic Rate (BMR) Calculations

TEE is calculated by estimating individual basal metabolic rate (BMR) showed in eq. (1) and (2) based on the Harris Benedict Equation formula [32].

$$BMR_{(gender=male)} = 66.5 + (13.75 \text{ x weight in kg}) + (5.003 \text{ x height in cm}) - (6.755 \text{ x age in years}) \quad 1$$

$$BMR_{(gender=female)} = 655.1 + (9.563 \text{ x weight in kg}) + (1.85 \text{ x height in cm}) - (4.676 \text{ x age in years} \quad 2$$

The estimated BMR value will then multiplied with a factor that correlates with user physical activity level to determine their total energy expenditure as illustrated in eq. (3) - (7). TEE value corresponds with individual daily kilocalories to maintain current body weight. Once the user indicated they want to lose weight, then SlimMe can easily calculate the number of calories needed to lose weight. The value will be reduced based on their desire weight goal per week (e.g. lose weight 0.25 kg per week equals with reducing 250 kcal intake, lose weight 0.5 kg per week equals with reducing 500 kcal intake).

$$TEE_{(PAL=\text{light to no exercise})} = BMR \text{ x } 1.2 \quad 3$$

$$TEE_{(PAL=\text{light exercise (1-3 days/week)})} = BMR \text{ x } 1.375 \quad 4$$

$$TEE_{(PAL=\text{ moderate exercise (3-5 days/week)})} = BMR \text{ x } 1.55 \quad 5$$

$$TEE_{(PAL=\text{ heavy exercise (6-7 days/week)})} = BMR \text{ x } 1.725 \quad 6$$

$$TEE_{(PAL=\text{ very heavy exercise (twice per day, extra heavy work out)})} = BMR \text{ x } 1.9 \quad 7$$
