# Supplementary material for: SlimMe, a Chatbot With Artificial Empathy for Personal Weight Management: System Design and Finding
Source: Front Nutr. 2022 Jun 23;9:870775. doi: 10.3389/fnut.2022.870775 (PMC9260382; doi:10.3389/fnut.2022.870775)
Supplement: Supplementary file 3 [file Data_Sheet_3.pdf]

Supplementary Material

Supplementary File 3. User Experience Evaluation Questionnaire after SlimMe 7-days simulation trials

QUESTIONNAIRE EVALUATION

SLIMME BOT 7-DAYS TRIALS

Dear SlimMe User,

We are conducting an external validation testing to find out how user will react to SlimMe chatbot and to evaluate the quality of the chatbot as well. SlimMe chatbot is a virtual assistant that can assist you during weight loss program by providing nutrition assessment, daily calorie intake and calorie burned estimation, daily reminder and motivational support. The idea is to emulate human conversation using conversational artificial intelligence (AI) through chat interfaces.

We would like to invite you to participate in this voluntary study by joining a 7 days trial to completing a certain task and interact with SlimMe bot through LINE messaging platform. However, you are not expected to benefit by participating in this study, and the study will not involve any discomfort. Although you can revoke your consent at any time, it will not be possible to extract and destroy your survey responses. Please note that the minimum retention period for data collection is five years.

Procedure

Before beginning the 7 days SlimMe Bot Trials, we will ask the participant to fill out a short questionnaire to collect information about the participant demographic characteristics, smartphone usage and familiarity with chatbot programs. After the trial session, we will ask the participant to answer a questionnaire for collecting data for SlimMe chatbot usability, effectiveness, satisfaction, and the perception of the chatbot performance.

Scenario

TASK	ITEM	EXPLANATION
SIMPLE	Greetings	Try to greet the bot
	Small Talk	Inform the bot that you want to lose weight, struggle with your diet, food pusher comment, say thank you, say bad appraisal
	Asking bot identity	Try to ask [what is chatbot] and [what it capable of], who develop the bot, etc
COMPLEX	Diet Goal	Select the diet plan offerred by the bot
	Nutritional Assessment	Inform your weight, gender, height, activity level, and weight goal
	Daily calorie need	Ask your daily calorie need
	User Profile	Check your inputted profile information and diet goal
VERY COMPLEX	Calorie Intake Tracker	Inform the bot what you eat and portion and ask the bot the calculate the calorie
	Calorie Burned Estimation	If you manage to do some exercise, ask the bot to estimate how many calories does it burn
	Calorie Log	Track your calorie need, intake, and burned from exercise

If you would like further information about this study, please contact:

Annisa Ristya Rahmanti (email: annisaristya@gmail.com)

Prof. Yu-Chuan (Jack) Li (email: jack@tmu.edu.tw)

Thank you very much for your participation

A. DEMOGRAPHIC

A01. What is your age?

- 18-24
- 25-34
- 35-44
- 45-54
- Above 54

A02. Which gender are you?

- Male
- Female

A03. Which academic field of study you identify most with? (You can select more than one option)

- Social sciences (Geography, Anthropology, Psychology, Economics, Political Sciences, etc)
- Natural Sciences (Physics, Chemistry, Biology, Earth Sciences, etc)
- Applied Mathematics and Statistics
- Computer Sciences
- Engineering Technology
- Medicine
- Oral Medicine
- Public Health
- Nutrition & Dietetics
- Nursing
- Linguistics & Language
- Literature & Arts
- Architecture-Design
- Other ______________

B. PHONE USAGE

B01. Do you regularly use a smartphone? (YES/NO)

B02. What smartphone's operating system do you currently use? (Check all that apply)

- o Android
- o Iphone
- o Windows Phone

B03. What are your 3 favorite apps for your smart phone?

- o Phone calls
- o Messaging platform
- o Shopping
- o Banking
- o Emailing
- o Exercising
- o Social networking (e.g., Facebook, Instagram)
- o Navigating with maps (e.g., finding a store)
- o Entertainment (e.g., movies, games)
- o Other ______________

B04. On average, how much time do you spend on your smartphone each day?

- o ≤ 1 hour
- o 2 – 5 hours
- o 6 – 9 hours
- o 10 – 12 hours
- o > 12 hours

B05. How much data per month do you use on your smartphone?

- 500mb ○ 1GB ○ 2GB ○ 3GB ○ 4GB ◉ 5GB ○ Over 5GB

B06. Do you check your phone within 5 minutes of waking up? (YES/NO)

B07. Have you ever used a chatbot before? (YES/NO)

C. PERFORMANCE

On a scale 1-5 (strongly disagree to strongly agree), how good would you say it is …………

Code	Items	1	2	3	4	5
C01	This chatbot understand what I said really well					
C02	The pace of interaction with the chatbot is appropriate					
C03	This chatbot work the way I expected it to					
C04	When interact with this chatbot, I am often experiencing slow response time					

D. USABILITY

On a scale 1-5 (strongly disagree to strongly agree), how good would you say it is …………

Code	Items	1	2	3	4	5
D01	The possibilities of this chatbot meet my requirements.					
D02	Using this chatbot is a frustrating experience					
D03	This chatbot is easy to use					
D04	I waste too much time on correcting things in this chatbot					

E. USEFULLNESS

On a scale 1-5 (strongly disagree to strongly agree), how good would you say it is …………

Code	Items	1	2	3	4	5
E01	Because of this chatbot, I can quickly track my calorie intake					
E02	Because of this chatbot, it is easily now for me to estimate my calorie burned from exercise					
E03	This chatbot makes it hard for me to track my daily calorie need					
E04	Because of this chatbot, I can effectively track my calorie intake					
E05	Because of this chatbot, I can effectively estimate my calorie burned from exercise					
E06	This chatbot is useless					

F. SATISFACTION

On a scale 1-5 (strongly disagree to strongly agree), how good would you say it is …………

Code	Items	1	2	3	4	5
F01	This chatbot is fun to use					
F02	I think I failed to understand some functionalities in this chatbot					
F03	I would recommend this chatbot to a friend					
F04	I am unsatisfied about this chatbot					

G. COMMENT/ FEEDBACK

G01. What did you think of this experience? Why (not)?

……………………………………………………………………………………………

…………………………………………..

G02. Do you think you would use this chatbot in real life? Why (not)?

…………………………………………………………………………………………………
…………………………………………..

G03. Do you think this chatbot is useful? Why (not)?

…………………………………………………………………………………………………
…………………………………………..

G04. Did you think there were functionalities missing in the chatbot? Which ones?

…………………………………………………………………………………………………
…………………………………………..
