# Supplementary material for: SlimMe, a Chatbot With Artificial Empathy for Personal Weight Management: System Design and Finding
Source: Front Nutr. 2022 Jun 23;9:870775. doi: 10.3389/fnut.2022.870775 (PMC9260382; doi:10.3389/fnut.2022.870775)
Supplement: Supplementary file 4 [file Data_Sheet_4.pdf]

Supplementary Material

Supplementary File 4. Descriptive summary of user agreement for the four-construct variables from user experience evaluation questionnaire (Note: 5-point likert scale (1-5) ranging from strongly disagree to strongly agree).

Code	Statement (Strongly disagree to strongly agree)	1	2	3	4	5
		N (%)	N (%)	N (%)	N (%)	N (%)
**Performance**						
C01	This chatbot understand what I said really well	-	1 (10)	3 (30)	6 (60)	-
C02	The pace of interaction with the chatbot is appropriate	-	-	4 (40)	6 (60)	-
C03	This chatbot work the way I expected it to	-	2 (20)	4 (40)	3 (30)	1 (10)
C04	When interact with this chatbot, I am often experiencing slow response time	1 (10)	2 (20)	2 (20)	4 (40)	1 (10)
**Usability**						
D01	The possibilities of this chatbot meet my requirements.	-	1 (10)	2 (20)	5 (50)	2 (20)
D02	Using this chatbot is a frustrating experience	3 (30)	3 (30)	3 (30)	1 (10)	-
D03	This chatbot is easy to use	-	-	2 (20)	6 (60)	2 (20)
D04	I waste too much time on correcting things in this chatbot	2 (20)	2 (20)	4 (40)	2 (20)	-
**Usefulness**						
E01	Because of this chatbot, I can quickly track my calorie intake	-	-	1 (10)	7 (70)	2 (20)
E02	Because of this chatbot, it is easier now for me to estimate my calorie burned from exercise	-	-	-	7 (70)	3 (10)
E03	This chatbot makes it hard for me to track my daily calorie need	3 (30)	3 (30)	1 (10)	3 (30)	-
E04	Because of this chatbot, I can effectively track my calorie intake	-	-	1 (10)	8 (80)	1 (10)
E05	Because of this chatbot, I can effectively estimate my calorie burned from exercise	-	-	1 (10)	8 (80)	1 (10)
E06	This chatbot is useless	7 (70)	2 (20)	1 (10)	-	-
**Satisfaction**						
F01	This chatbot is fun to use	-	-	-	6 (60)	4 (40)
F02	I think I failed to understand some functionalities in this chatbot	2 (20)	4 (40)	3 (30)	1 (10)	-
F03	I would recommend this chatbot to a friend	-	-	1 (10)	7 (70)	2 (20)
F04	I am unsatisfied about this chatbot	3 (30)	4 (40)	2 (20)	1 (10)	-
